# Neighborhood greenspace exposure as a protective factor in dementia risk among U.S. adults 75 years or older: a cohort study

**DOI:** 10.1186/s12940-022-00830-6

**Published:** 2022-01-15

**Authors:** Erik D. Slawsky, Anjum Hajat, Isaac C. Rhew, Helen Russette, Erin O. Semmens, Joel D. Kaufman, Cindy S. Leary, Annette L. Fitzpatrick

**Affiliations:** 1 University of Washington, Seattle, WA USA; 2 Johns Hopkins University, Baltimore, MD USA; 3 University of Montana, Missoula, MT USA

**Keywords:** Dementia, Greenspace, NDVI, Residential exposures, Alzheimer’s disease

## Abstract

**Background:**

Research suggests that greenspace may confer neurocognitive benefits. This study examines whether residential greenspace is associated with risk of dementia among older adults.

**Methods:**

Greenspace exposure was computed for 3047 participants aged 75 years and older enrolled in the Gingko Evaluation of Memory Study (GEMS) across four U.S. sites that prospectively evaluated dementia and its subtypes, Alzheimer’s disease (AD), vascular dementia (VaD), and mixed pathologies, using neuropsychiatric evaluations between 2000 and 2008. After geocoding participant residences at baseline, three greenspace metrics—Normalized Difference Vegetative Index, percent park overlap within a 2-km radius, and linear distance to nearest park—were combined to create a composite residential greenspace measure categorized into tertiles. Cox proportional hazards models estimated the associations between baseline greenspace and risk of incident all-cause dementia, AD, and Mixed/VaD.

**Results:**

Compared to low residential greenspace, high residential greenspace was associated with a reduced risk of dementia (HR = 0.76 95% CI: 0.59,0.98) in models adjusted for multiple covariates. After additional adjustment for behavioral characteristics, Apolipoprotein E ɛ4 status, and other covariates, the association was slightly attenuated (HR = 0.82; 95% CI:0.63,1.06). Those exposed to medium levels of greenspace also had 28% lower risk (HR = 0.72; CI: 0.55, 0.95) of dementia compared to those with low greenspace in adjusted models. Subtype associations between high residential greenspace and AD were not statistically significant. Greenspace was not found to be significantly associated with mixed/vascular pathologies.

**Conclusions:**

This study showed evidence for an association between residential greenspace and all-cause dementia among older adults. Future research with larger sample size, precise characterization of different dementia subtypes, and assessment of residential greenspace earlier in life may help clarify the role between exposure to greenspace and dementia risk.

**Supplementary Information:**

The online version contains supplementary material available at 10.1186/s12940-022-00830-6.

## Abstract (article highlights)

- Residential greenspace exposure was found to be associated with moderate cognitive health benefit for older U.S. adults.
- Dementia subtypes may be differentially impacted by exposure to residential greenspace.
- Combining multiple greenspace measures may better capture a holistic picture of exposure to greenspace compared to one measure alone.

**Supplementary Information:**

The online version contains supplementary material available at 10.1186/s12940-022-00830-6.

## Background

Alzheimer’s Disease and Related Dementias (ADRD) are estimated to globally cost over 2 trillion dollars by 2030 [1]. A recent study projected a two-fold increase in ADRD burden from 1.6% of the U.S. population in 2014 to 3.3% by 2060 with 13.9 million Americans suffering from ADRD [2]. It has been suggested that 35% of dementia cases worldwide could be prevented by addressing modifiable risk factors, with 15% of cases addressable during later life (> 65 years) [3]. There has been increasing interest in exposure to greenspace as a possible modifiable protective factor because it may improve cognitive domains [4–6], reduce stress [7–9], increase physical exercise [10], improve social ties [11, 12] and mitigate air pollution [13–15].

As the mechanisms underlying dementia continue to be elucidated, it is becoming clear that chronic inflammation and stress play important roles in various dementia pathologies [16–18]. Greenspace as an exposure may play a role in preventing or mitigating such risk factors for dementia [19–21]. Studies have found greenspace exposure to be associated with healthier cortisol profiles [22, 23], which is a known biomarker for stress. Furthermore, greenspace may impact mental health and wellbeing more broadly [24–30]. Lastly, environments with abundant greenspace may promote physical activity which has been shown to reduce ADRD risk [31]. Yet, it is still unclear how exposure to greenspace influences risk of dementia among older adults, particularly risk of subtypes like vascular dementia (VaD), Alzheimer’s disease (AD), and mixed pathologies. Examination of subtypes may aid in elucidating possible mechanisms of action (i.e. physical activity, pollution mitigation, stress reduction, social contact) for the greenspace-dementia relationship. Some research has already noted inverse associations between elevated greenspace exposure and AD using an ecological design [32].

This work aims to evaluate whether neighborhood greenspace is associated with reduced risk of incident dementia, including all-cause dementia and its subtypes of AD and Mixed/VaD, using a well characterized study of older adults evaluated for dementia over eight years.

## Methods

### Study data

The Ginkgo Evaluation of Memory Study (GEMS) was a double-blind, placebo controlled clinical trial developed to investigate the effect of *Gingko biloba* on dementia and its subtypes in older adults [33]. Participants were randomized using a block design to either twice-daily doses of 120 mg *G. biloba* extract or an identical appearing placebo [34, 35]. The original trial ran from 2000 to 2008 with more than 3000 participants from four sites: Winston-Salem, North Carolina; Hagerstown, Maryland; Pittsburgh, Pennsylvania; and Sacramento, California with a median follow-up time of 6.1 years. Participants were adults 75 years of age or older at baseline, did not have dementia at enrollment, and had provided a home address. Participants were assessed every six months for up to 8 years. Data were collected by physical examination, blood testing, and survey questionnaires. For our analysis 3069 participants had residential address information available; 22 of those addresses could not be geocoded bringing our sample to 3047 GEMS participants.

### Outcomes

The classification of dementia was based on DSM-IV criteria [36]. Dementia due to AD was determined using criteria from the National Institute of Neurological and Communicative Disorders and Stroke/Alzheimer’s Disease and Related Disorders Association (NINCDS-ADRDA) [37]. Dementia assessment was extremely robust in the GEMS trial, using a combination of Global Clinical Dementia Rating (CDR) [38], Alzheimer’s Disease Assessment Scale (ADAS) [39], and Modified Mini-Mental State Exam (3MS) [40] to screen participants and a series of 12 neurophysiological tests in six domains to determine dementia and subtype status during follow-up [33]. Participants were administered this full neuropsychological battery at baseline. Every six months thereafter, all participants were re-assessed with the cognitive screening instruments to determine if the full battery of dementia assessments should be re-administered. For participants whose scores dropped a pre-specified number of points, dependent on individual participant baseline scores for two of the three tests (3MS CDR, or ADAS), the participant proxy reported new cognitive issues, a private physician diagnosed dementia, or the participant was prescribed a medication for dementia, the full battery was repeated. A blinded panel of experts consisting of two neurologists, two neuropsychologists, and one psychometrician reviewed results of the full battery. Participants were then classified as having dementia and its subtypes of AD only, AD and VaD (mixed), VaD only, or other type of dementia according to criteria from the NINCDS-ADRDA, National Institute of Neurological Disorders and Stroke-Association, Internationale pour le Recherche et l’Enseignement en Neurosciences, and the Alzheimer’s Disease Diagnostic and Treatment Centers. Individuals were classified as either having dementia or not by end of study. In total, 523 cases of dementia occurred during the GEMS trial. Secondary analyses to evaluate AD only and Mixed/VaD dementia using criteria described above were also conducted. Of the sample with geocoded data, 518 cases of dementia were available for analysis.

### Greenspace measures

Three data sources, the United States Geologic Survey Protected Areas Databases (USGS-PAD) as of 2018 [41], Trust for Public Land (ParkServe) as of 2018 [42], and eMODIS Normalized Difference Vegetation Index (NDVI) average for 2001 [43] were combined to create a composite metric for residential greenspace exposure. Each of these data sources define and measure greenspace exposure. USGS-PAD provides a standardized database for all federally recognized protected areas, which commonly includes parks and other forms of greenspaces that enable human-environment interaction. Smaller non-federally recognized greenspaces, like school yards and boulevards are captured by ParkServe. The combination of these two data sources yielded over 70,000 bounded areas across the four study cities. However, not all greenspaces are formally bounded areas, and thus NDVI can add critical information about the overall density of vegetation surrounding a residence regardless of cartographic boundaries. Seven-day composite summer values were used to provide an estimate of the maximum possible level of “greenness” to which a participant may have been exposed. Additionally, validation of NDVI as a neighborhood greenness measure was confirmed in a study that found a strong correlation with environmental psychologists’ ratings of neighborhood greenness via photographs taken near residences to examine vegetative density [44], making NDVI a useful tool for assessing neighborhood greenness. Three essential measures were taken to determine a residence’s level of greenspace exposure. First, linear distance from the residence to the nearest park centroid was recorded in meters (m). Second, radial buffers were created out to 2 km from the residence in 500-m increments. The area of park overlap was then calculated for each buffer with cumulative totals calculated as a percent of the area of the buffer. Last, 250 m resolution NDVI pixel values were transformed to standardized − 1 to 1 spectrum values, with values closer to 1 indicating more living vegetation. Mean values were calculated within the 2000 m buffer to create a single average NDVI value for each residence. These three measures were standardized as z-scores with a mean of 0 and a standard deviation (SD) of 1. Distance to the nearest park centroid was reverse coded so that larger values would be associated with closer access to park, just as larger values of NDVI and percent park overlap are also associated with more greenspace exposure. All measures were collected from the GEMS participant’s address provided at baseline. The three standardized scores were then combined into a single composite greenness score by taking the average across all three standardized scores. Participants were then grouped into low, medium, and high greenspace based on percentile. Those with scores in the 33^rd^percentile and below are classified as low exposure, 34th to 66th percentiles are classified as medium exposure, and those with scores in the 67th percentile and above are classified as high exposure. Utilizing a composite measure attempts to capture a more holistic picture of greenspace exposure. Sensitivity analyses were conducted to compare the composite measure the standardized single metrics (Supplemental Table 1).

### Covariates

Selected covariates were included in the analysis to account for potential confounding and to test for effect modification. Demographic variables included age (in years) at randomization, sex (male or female), race (White, People of Color) as a social construct that may account for potential differences specific to race, treatment arm (*G.biloba* or placebo), and recruitment site (Winston-Salem, North Carolina; Hagerstown, Maryland; Pittsburgh, Pennsylvania; and Sacramento, California). Additional health behavior covariates included body mass index (BMI as a continuous scale), smoking status (measured by number of pack years, passive smoking percentage, and current smoking status defined as current former or never), alcohol consumption (number of drinks per week on average), and Assessment of Activities of Daily Life (ADL) mobility score which provides a standardized method of evaluating disability [45] by self-report questionnaire based on difficulty with specific mobility and strength related tasks. Lastly, we included Apolipoprotein E ɛ4 (APOE ɛ4) genotype (i.e., presence of at least one copy of the ɛ4 allele), and neighborhood socioeconomic status (NSES) scores where values under zero represent lower status and values above zero represent higher status. NSES was assessed with a similar index used in the Multi-Ethnic Study of Atherosclerosis [46] with values based on residential address at the census tract level. Principal components analysis of Census 2000 summary files and 2005–2009 American Community Survey indicated the following seven variables had the largest loadings and were used to construct NSES: % high school education, % with Bachelor’s degree, % with managerial occupation, median home value, % with interest/dividend/rental income, median household income, % of households with income >$50,000, and binary rurality assessed via Rural Urban Commuting Area codes of census tracts containing baseline addresses (codes of 3 or less is defined as urban and 4 to 10 are defined as rural) [47].

### Statistical analysis

Analyses were conducted using R Version 4.0.1 [48] and QGIS 3.14 [49]. To estimate the association between greenspace and time until dementia diagnosis, Cox proportional hazards regression was used. Greenspace categories were included as dummy variables with low greenspace being the reference group. Covariates were added to regression models in a hierarchical fashion beginning with a minimally adjusted model and concluding in a fully adjusted model that included several behavioral risk factors and APOE ɛ4 status. Model A adjusted for a minimal set of confounders: year, race, sex, treatment arm, and recruitment site. Model B further included NSES and education, as socioeconomic status (SES) is thought to confound the association between environmental exposures and health outcomes [50]. Model C added ADL mobility, MCI at baseline status, BMI, and APOE ɛ4 status. Lastly, model D included a measure of rurality using rural-urban commuting codes to address possible urban/rural confounding. Cox models provide a flexible approach for assessing multiple predictors of right-censored time-to-event outcomes. Our models were run using Breslow’s method of approximation for ties in the time data. We tested for the proportional hazards assumption using Schoenfeld residuals with all-cause dementia models A, B, C, and D having global test *p* values of 0.77, 0.82, 0.32, and 0.36 respectively. Models assessing subtypes were run with the same covariate adjustment scheme listed above.

### Multiple imputation

The allele status for APOE ɛ4 is a strong risk factor for AD [51]. This is an important precision variable for analysis, but 615 (20.2%) of the GEMS cohort were not tested for the gene. To account for missing APOE ɛ4 status as well as missingness for all other study variables, we used Multiple Imputation by Chained Equations (mice) to impute missing APOE ɛ4 and other study variable values [52]. Through the mice procedure, we generated 10 imputed datasets of missing observations for all variables. Models were run within each of the 10 datasets, and we pooled the parameter estimates and standard errors based on Rubin’s rules [53].

## Results

Our study sample consisted of 3047 participants. A total of 518 were classified with dementia over the course of the study. The GEMS cohort was predominantly White (95%) and majority male (54%). Mean age at baseline was 78.6 years (Table 1). Participants were spread across the four study sites with many participants receiving a college degree (23%). Average BMI was 27.13 kg/m^2^. Alcohol consumption was common among this sample with 74% reporting some form of weekly consumption. Our study sample, despite advanced age, was largely independently mobile (69%). Additionally, 24% of those tested had the APOE ɛ4 allele. Among dementia cases, Alzheimer’s Disease (AD) was the most commonly identified subtype. Table 2 shows the distribution of greenspace metrics by exposure group as well as information on dementia subtypes.

**Table 1:** GEMS participant characteristics by greenspace exposure group presented as n and percent or mean and standard deviation

	Greenspace Exposure Group^a^				
	Low	Med	High	Total	***p***-value^*^
**n (%)**	1017 (33)	1014 (33)	1016 (33)	3047	
**Age in years (mean (SD))**	78.7 (3.35)	78.8 (3.34)	78.4 (3.13)	78.6 (3.28)	0.01
**Dementia**	205 (20.2)	169 (16.7)	144 (14.2)	518 (17.0)	< 0.01
**Race (% self-identified White)**	964 (94.8)	972 (95.9)	972 (95.7)	2908 (95.4)	0.47
**Sex (% male)**	552 (54.3)	534 (52.7)	550 (54.1)	1636 (53.7)	0.72
**Recruitment site**					< 0.01
Winston-Salem, NC	301 (29.6)	282 (27.8)	149 (14.7)	732 (24.0)	
Sacramento, CA	527 (51.8)	295 (29.1)	81 (8.0)	903 (29.6)	
Hagerstown, MD	75 (7.4)	224 (22.1)	148 (14.6)	447 (14.7)	
Pittsburg, PA	114 (11.2)	213 (21.0)	638 (62.8)	965 (31.7)	
**Treatment group (% Ginkgo)**	519 (51.0)	513 (50.6)	498 (49.1)	1530 (50.2)	0.66
**Education**					0.32
No high school diploma	367 (36.1)	348 (34.3)	379 (37.3)	1094 (35.9)	
High school diploma	269 (26.5)	265 (26.1)	236 (23.2)	770 (25.3)	
Some college	146 (14.4)	159 (15.7)	175 (17.2)	480 (15.8)	
College graduate	235 (23.1)	242 (23.9)	226 (22.2)	703 (23.1)	
**BMI in** kg/m^2^ **(mean(SD))**	27.2 (4.24)	27.1 (4.24)	27.1 (4.40)	27.1 (4.29)	0.98
**Alcohol Consumption (mean(SD))**^b^	3.71 (6.56)	3.49 (6.60)	3.31 (6.28)	3.50 (6.48)	0.38
**Smoking pack years (%)**					0.34
0	390 (41.5)	418 (44.8)	424 (44.8)	1232 (43.7)	
> 0 - ≤24	289 (30.7)	265 (28.4)	254 (26.8)	808 (28.7)	
> 24	261 (27.8)	250 (26.8)	268 (28.3)	779 (27.6)	
**APOE ɛ4 (% allele present)**	186 (23.8)	198 (24.4)	189 (22.5)	573 (23.6)	0.64
**ADL mobility (% independent)**	700 (68.9)	689 (67.9)	702 (69.1)	2091 (68.6)	0.39
**MCI at baseline**	178 (17.5)	161 (15.9)	137 (13.5)	476 (15.6)	0.043
**NSES (mean(SD))**	0.70 (3.15)	−0.19 (3.14)	− 0.61 (3.10)	− 0.03 (3.18)	< 0.01
**Rural**^c^	73 (7.2)	50 (4.9)	41 (4.0)	164 (5.4)	< 0.01

*Abbreviations*: *ADL* activities of daily life, *APOE ɛ4* apolipoprotein E ɛ4, *BMI* body mass index, *MCI* mild

cognitive impairment; NSES, neighborhood socioeconomic status

^*^:*P*-value reflects results from ANOVA/chi-square between greenspace exposure groups and covariate

^a^: Low: ≤33%, Med:34–66%, High:≥67

^b^: Average number of drinks per week

^c^: Binary rural based on Rural Urban Community Area codes

**Table 2:** Greenspace exposure groups and dementia subtypes presented as n and percent or mean and standard deviation

	Greenspace Exposure Group				
	Low	Med	High	Total	p^*^
**n**	1017	1014	1016	3047	
**Dementia (%)**	205 (20.2)	169 (16.7)	144 (14.2)	518 (17.0)	< 0.01
**AD only (%)**	138 (13.6)	110 (10.8)	102 (10.0)	350 (11.5)	0.03
**Mixed/VaD (%)**	58 (5.7)	49 (4.8)	39 (3.8)	146 (4.8)	0.14
**Mean (SD)**	**Greenspace Metrics**				
**Distance (m)**	1789 (2699)	907 (750)	780 (522)	1159 (1706)	< 0.01
**Mean percent Overlap**	0.94 (1.47)	1.49 (2.26)	6.69 (11.05)	3.04 (7.06)	< 0.01
**NDVI**	0.46 (0.11)	0.57 (0.08)	0.68 (0.09)	0.57 (0.13)	< 0.01
**Composite**	−0.26 (0.71)	0.12 (0.31)	0.38 (0.53)	0.00 (0.60)	< 0.01

*Abbreviations*: *AD* Alzheimer’s disease, *m* linear meters, *NDVI* normalized differences vegetative index, *SD* standard deviation, *VaD* Vascular dementia

^*^:*P*-value reflects results from ANOVA/ chi-square between green space exposure group

**Fig. 1:**
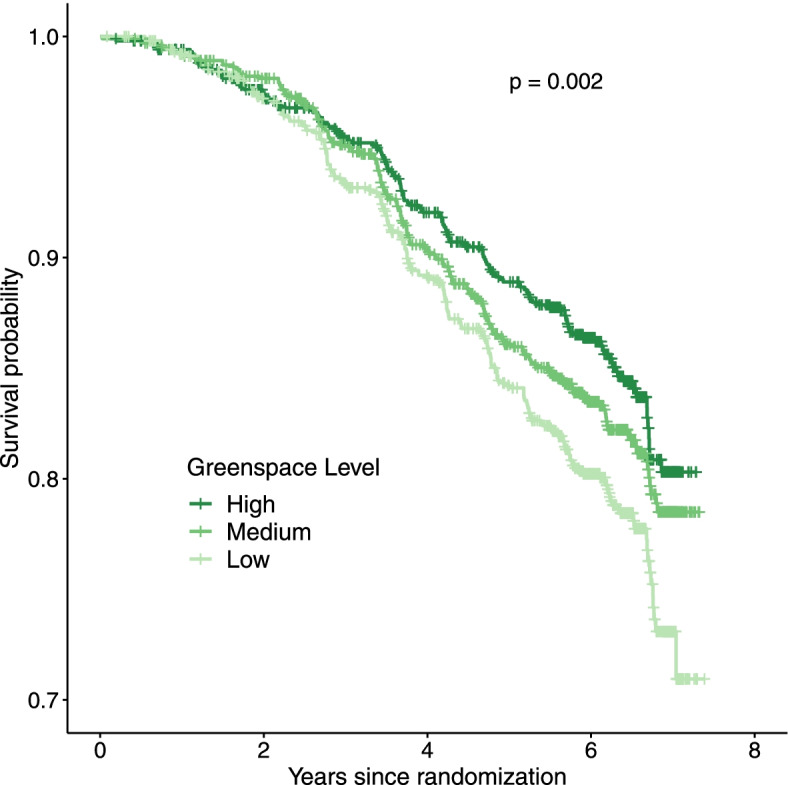
Kaplan-Meier plot for dementia by residential greenspace group

As reflected in the Kaplan-Meier plot (Fig. 1) and the minimally adjusted Model A (Table 3), those living in high greenspace areas compared to low showed a 24% reduction in risk of dementia (Hazard Ratio [HR] = 0.76; 95% CI: 0.59,0.97). These findings remained consistent in Model B after the addition of NSES and education (HR = 0.76; 95% CI: 0.59,0.98). With the addition of all covariates in Model D, this association was attenuated and no longer statistically significant (HR = 0.82; 95% CI: 0.63,1.06) but still indicated a substantially lower point estimate. Medium greenspace exposure was consistent across all models and did remain statistically significant in Model D (HR = 0.77; 95% CI: 0.62,0.96).

**Table 3:** Hazard ratios and 95% confidence intervals from Cox proportional hazard models for association between composite greenspace exposure dementia and dementia subtypes with multiple imputation

	Greenspace Exposure Group		
	Low	Med	High
**All-cause dementia**			
**Model A**	REF	0.77 (0.62,0.96)	0.76 (0.59,0.97)
**Model B**	REF	0.77 (0.62,0.96)	0.76 (0.59,0.98)
**Model C**	REF	0.77 (0.62,0.95)	0.82 (0.63,1.06)
**Model D**	REF	0.77 (0.62,0.96)	0.82 (0.63,1.06)
**Mixed/VaD**			
**Model A**	REF	0.80 (0.54,1.18)	0.72 (0.46,1.13)
**Model B**	REF	0.83 (0.56,1.24)	0.77 (0.48,1.23)
**Model C**	REF	0.84 (0.56,1.24)	0.83 (0.52,1.32)
**Model D**	REF	0.84 (0.56,1.24)	0.83 (0.52,1.33)
**Alzheimer’s Disease only**			
**Model A**	REF	0.74 (0.56,0.97)	0.80 (0.59,1.09)
**Model B**	REF	0.72 (0.55,0.94)	0.78 (0.57,1.06)
**Model C**	REF	0.72 (0.55,0.95)	0.84 (0.61,1.16)
**Model D**	REF	0.73 (0.56,0.96)	0.84 (0.61,1.16)

Model A: adjusted for year, race, sex, treatment arm, site

Model B: adjusted for Model A + NSES, education

Model C: adjusted for Model B + mobility, MCI at baseline, alcohol, smoking, APOE ɛ4, BMI

Model D: adjusted for Model C + rurality

Further analyses examined differential impact of greenspace exposure by dementia subtype (Table 3). In comparison to those with low greenspace, those with high greenspace exposure had a reported HR of 0.72 (CI: 0.46, 1.13) for Mixed/VaD in Model A. Although the HR suggested a 28% reduction in risk, this association was not statistically significant and remained so after adjusting for additional covariates. For the Alzheimer’s disease subtype, those with medium greenspace exposure had 26% lower risk (HR = 0.74; CI: 0.56, 0.97) compared to low greenspace in Model A. There was no statistically significant difference for the high greenspace exposure group in the fully adjusted model. Point estimates, however, indicate lower hazard ratios for all subtypes. Sensitivity analyses comparing the composite greenspace to standardized individual measures indicated that the composite measure produces stronger and more precise estimates for all cause dementia and Alzheimer’s disease. NDVI is more strongly related to Mixed/VaD than the composite metric (Supplemental Table 1).

## Discussion

This study evaluated associations between residential greenspace exposure and risk of dementia among older adults. By combining multiple spatial data sources, we were able to create a composite measure that captured multiple dimensions of greenspace and may better represent total greenspace exposure. Additionally, we applied this composite metric to a robust and well characterized cohort specifically designed to evaluate dementia and its subtypes. We observed protective associations between high greenspace exposure and all-cause dementia risk in models. After adjusting for socio-demographic covariates and further adjusting for additional behavioral characteristics, these associations were somewhat attenuated. Our results suggest that greenspace may offer protective benefits to those with medium to high residential exposure.

Results from this study appear to be generally consistent with other findings showing modest associations between greenspace and dementia outcomes [54–56]. Without more detailed information it is difficult to parse out whether mediation by physical exercise, air pollution mitigation, cognitive restoration, or increased socialization are the main route by which greenspace may be lowering risk or if it is a combination of many routes. Our high greenspace exposure group had the fewest dementia cases (Table 2), which might indicate the efficacy of greenspace for reducing dementia risk. A recent study from Canada which used a large national dataset found statistically significant associations with urban greenspace exposure, dementia, and stroke [54]. Other studies have evaluated individual components of greenspace exposure, like NDVI, finding protective effects among older adults and Alzheimer’s risk [57]. Using a large Medicare dataset, findings showed statistically significant but modest associations. Studies that evaluated greenspace exposure among a smaller sample often resulted in non-statistically significant findings [58, 59]. It was noteworthy that associations of medium greenspace exposure with dementia outcomes were similar or even sometimes stronger in magnitude than those of high greenspace exposure. This may, in part, reflect a maximum threshold of greenspace benefit. Beyond a certain level of greenness additional greenness may not confer added benefit, particularly for an older and less active population [60, 61]. This is most apparent in the AD findings, where medium, but not high, greenspace exposure was significantly associated with all-cause dementia and AD.

While the mechanisms of various forms of dementia are still being elucidated, it is worth noting that greenspace may not influence each subtype of dementia in the same way. The Mixed/VaD subtypes may be more heavily influenced by exposures that impact cardiovascular health. While greenspace has been shown to mitigate the impacts of air pollution, further research is needed to determine if greenspace significantly alters air pollution effects for dementia outcomes [62]. Furthermore, countries with large national registries or insurance databases may add to our ability to study the relationship between greenspace and dementia [54]. Such future work may be warranted particularly considering research showing improvements in a variety of cognitive and physical outcomes is associated with individual and community-level interventions involving increasing greenspace exposure [63–67]. These promising interventions are perhaps the strongest evidence for the effects of greenspace exposure on dementia and cognitive outcomes.

Our study, like many greenspace studies, was limited in its ability to accurately identify and map greenspaces in which people may interact with the natural environment. This was partially ameliorated by the inclusion of several different sources of greenspace information but remains a perpetual concern. Further, we only assessed greenspace exposure at baseline, and could not account for additional features, amenities, safety, aesthetics, access, or other aspects that may impact space use. As a study of residential greenspace exposure there is also the possibility that the greenness around a residence does not reflect the level of exposure experienced by the participant as part of their routine activity spaces. Most of our sample was independently mobile (68.6%), but mobility for the remainder of the sample and among older adults more generally, would limit time spent outdoors, this makes it difficult to measure physical activity mediated greenspace exposure. We also have no information on their exposure to greenspace earlier in life, which may have a larger impact on cognitive health than later life greenspace exposure [61] and rely on baseline addresses for determination of greenspace exposure. Additionally, the study sample may possibly be uniquely robust in their survival and absence of dementia at 75, and thus not as readily susceptible to environmental influences on dementia risk. The lack of racial and economic diversity within our study population may be partly attributed to historical neighborhood discrimination. It is well characterized that residing in neighborhoods with no or low greenness is associated with increased risk of mortality among older adults [68, 69]. Lastly, concerns of selection and generalizability exist due to the lack of racial, economic, and educational diversity in this sample. The GEMS sample was predominantly White, greatly reducing our ability to assess disparity in exposure and outcome and determine impacts from stressors related to lived experiences and racial discrimination [70]. Furthermore, the highly educated make-up of our sample has implications for generalizability given the importance of education in dementia development [71]. Our study benefitted from several strengths. Chief among them are the well characterized GEMS cohort, the robust dementia assessment, and the relatively long follow-up period. The multiple greenspace databases also allowed us to include over 70,000 bounded areas and when combined with NDVI create a more holistic picture of the greenspace exposure beyond parks/urban greenspace or vegetative cover alone.

Future greenspace research should focus on understanding which types of greenspace and what activities occur in them that may confer health benefits. This could involve modern data collection methodologies such as GPS-monitoring which has been utilized in some recent studies in order to capture exposure to greenspace beyond one’s residential environment [21]. This type of study may assist in our understanding of potential mechanisms by which greenspace may be improving health including cognition. This study’s findings may inform additional longitudinal analyses with the GEMS cohort to characterize specific subtypes of dementia and assess greenspace exposure at earlier times in the participant’s life.

## Conclusions

This study of residential greenspace exposure and dementia risk among older U.S. adults found evidence for protective associations between elevated greenspace exposure and incident dementia. Concerns over the aging U.S. population and possible interventions continue to grow. Larger and more robust investigations of how older adults interact with greenspaces may be warranted to accurately understand the role residential greenspace exposure plays in dementia risk among older adults.

## Supplementary Information

**Additional file 1: Supplemental Table 1.** Hazard ratios and 95% confidence intervals from Cox proportional hazard models for associations between composite greenspace exposure, standardized single greenspace exposure dementia and dementia subtype with multiple imputation.

## Data Availability

Data collected for this analysis includes identifying information on participants, whose participation was confidential, and hence cannot be released in complete form. The study protocol, statistical analysis plan, and informed consent forms are available on request. De-identified data on the GEMS study, including a data dictionary, are available through the National Centralized Repository for Alzheimer’s Disease and Related Dementias at ncrad.iu.edu.
