# Supplementary material for: Neighborhood greenspace exposure as a protective factor in dementia risk among U.S. adults 75 years or older: a cohort study
Source: Environ Health. 2022 Jan 15;21:14. doi: 10.1186/s12940-022-00830-6 (PMC8760791; doi:10.1186/s12940-022-00830-6)
Supplement: Supplementary file 1 — Additional file 1: Supplemental Table 1. Hazard ratios and 95% confidence intervals from Cox proportional hazard models for associations between composite greenspace exposure, standardized single greenspace exposure dementia and dementia subtype with multiple imputation. [file 12940_2022_830_MOESM1_ESM.docx]

*Supplemental Table 1: Hazard ratios and 95% confidence intervals from Cox proportional hazard models for associations between composite greenspace exposure, standardized single greenspace exposure dementia and dementia subtype with multiple imputation.*

|  | **Greenspace Exposure Group^a^** | | |
| --- | --- | --- | --- |
|  | **Low** | **Med** | **High** |
| **All-cause dementia** | | | |
| **Model A - composite** | REF | 0.77 (0.62­­,0.96) | 0.76 (0.59­­,0.97) |
| Model A - zNDVI | REF | 0.84 (0.67, 1.06) | 0.79 (0.61,1.03) |
| Model A - z%Overlap | REF | 1.05 (0.85, 1.29) | 0.96 (0.77,1.18) |
| Model A - zDistance | REF | 1.03 (0.84, 1.29) | 0.95 (0.77,1.19) |
| **Model B - composite** | REF | 0.77 (0.62­­,0.96) | 0.76 (0.59­­,0.98) |
| Model B - zNDVI | REF | 0.84 (0.67, 1.06) | 0.79 (0.60,1.03) |
| Model B - z%Overlap | REF | 1.05 (0.85, 1.29) | 0.96 (0.78,1.18) |
| Model B - zDistance | REF | 1.04 (0.85, 1.29) | 0.96 (0.77,1.19) |
| **Model C - composite** | REF | 0.77 (0.62­­,0.96) | 0.82 (0.63­­,1.06) |
| Model C - zNDVI | REF | 0.82 (0.65, 1.03) | 0.82 (0.62,1.07) |
| Model C - z%Overlap | REF | 1.10 (0.89, 1.36) | 0.99 (0.80,1.23) |
| Model C - zDistance | REF | 1.12 (0.90, 1.38) | 0.98 (0.78,1.22) |
| **Model D - composite** | REF | 0.77 (0.62­­,0.96) | 0.82 (0.63­­,1.06) |
| Model D - zNDVI | REF | 0.81 (0.64, 1.02) | 0.80 (0.61,1.06) |
| Model D - z%Overlap | REF | 1.11 (0.90, 1.38) | 1.00 (0.80,1.24) |
| Model D - zDistance | REF | 1.13 (0.91, 1.40) | 0.99 (0.79, 1.24) |
| **Mixed/VaD** | | | |
| **Model A - composite** | REF | 0.80 (0.54­­,1.18) | 0.72 (0.46­­,1.13) |
| Model A - zNDVI | REF | 0.55 (0.35, 0.88) | 0.76 (0.47,1.24) |
| Model A - z%Overlap | REF | 1.00 (0.67, 1.49) | 0.97 (0.66,1.43) |
| Model A - zDistance | REF | 0.79 (0.52, 1.18) | 0.92 (0.62,1.19) |
| **Model B - composite** | REF | 0.83 (0.56­­,1.24) | 0.77 (0.48­­,1.23) |
| Model B - zNDVI | REF | 0.58 (0.37, 0.93) | 0.80 (0.49,1.29) |
| Model B - z%Overlap | REF | 0.98 (0.66, 1.45) | 1.00 (0.66,1.43) |
| Model B - zDistance | REF | 0.81 (0.54, 1.21) | 0.91 (0.62,1.36) |
| **Model C - composite** | REF | 0.84 (0.56­­,1.24) | 0.83 (0.52­­,1.32) |
| Model C - zNDVI | REF | 0.58 (0.37, 0.91) | 0.82 (0.51,1.32) |
| Model C - z%Overlap | REF | 1.02 (0.68, 1.52) | 1.04 (0.70,1.54) |
| Model C - zDistance | REF | 0.83 (0.55, 1.26) | 0.92 (0.61,1.38) |
| **Model D - composite** | REF | 0.84 (0.56­­,1.24) | 0.83 (0.52­­,1.33) |
| Model D - zNDVI | REF | 0.58 (0.36, 0.91) | 0.82 (0.51,1.33) |
| Model D - z%Overlap | REF | 1.01 (0.68, 1.51) | 1.03 (0.70,1.53) |
| Model D - zDistance | REF | 0.83 (0.55, 1.25) | 0.91 (0.60,1.37) |
| **Alzheimer's Disease only** | | | |
| **Model A - composite** | REF | 0.74 (0.56­­,0.97) | 0.80 (0.59­­,1.09) |
| Model A - zNDVI | REF | 0.99 (0.75, 1.31) | 0.86 (0.61,1.19) |
| Model A - z%Overlap | REF | 1.04 (0.81, 1.34) | 0.95 (0.73,1.18) |
| Model A - zDistance | REF | 1.09 (0.84, 1.40) | 0.96 (0.74,1.25) |
| **Model B - composite** | REF | 0.72 (0.55­­,0.94) | 0.78 (0.57­­,1.06) |
| Model B - zNDVI | REF | 0.97 (0.73, 1.29) | 0.84 (0.60,1.17) |
| Model B - z%Overlap | REF | 1.04 (0.81, 1.34) | 0.94 (0.73,1.22) |
| Model B - zDistance | REF | 1.09 (0.85, 1.41) | 0.97 (0.75,1.26) |
| **Model C - composite** | REF | 0.73 (0.55­­,0.95) | 0.84 (0.61­­,1.16) |
| Model C - zNDVI | REF | 0.93 (0.70, 1.24) | 0.88 (0.62,1.24) |
| Model C - z%Overlap | REF | 1.10 (0.85, 1.44) | 0.97 (0.74,1.27) |
| Model C - zDistance | REF | 1.18 (0.91, 1.53) | 0.99 (0.75,1.30) |
| **Model D - composite** | REF | 0.73 (0.56­­,0.96) | 0.84 (0.61­­,1.16) |
| Model D - zNDVI | REF | 0.92 (0.69, 1.22) | 0.86 (0.61,1.22) |
| Model D - z%Overlap | REF | 1.12 (0.86, 1.45) | 0.98 (0.75,1.28) |
| Model D - zDistance | REF | 1.19 (0.92, 1.55) | 1.01 (0.76,1.33) |

^a^: z-measures represent the standardized (z-scored) greenspace metrics.

| zNDVI: Average summer 2001 NDVI value for 2000m buffer around residence |
| --- |
| z%Overlap: Percent of park area overlapping 2000m buffer around residence |
| zDistance: Reverse coded linear distance in meters to the nearest park centroid from residence |

Model A: adjusted for year, race, sex, treatment arm, site

Model B: adjusted for Model A + NSES, education

Model C: adjusted for Model B + mobility, MCI at baseline, alcohol, smoking, APOE ɛ4, BMI

Model D: adjusted for Model C + rurality
